# Association Between Posterior Tibial Slope and Clinical Outcomes After Isolated Anterior Cruciate Ligament Reconstructions

**DOI:** 10.7759/cureus.46679

**Published:** 2023-10-08

**Authors:** Ugur Kasman, Serkan Surucu, Ozgur Korkmaz

**Affiliations:** 1 Department of Orthopedics and Traumatology, Bahçeşehir University School of Medicine, Istanbul, TUR; 2 Orthopedics and Rehabilitation, Yale University, New Haven, USA

**Keywords:** tegner lysholm knee scoring system, arthroscopy knee, anterior cruciate ligament (acl) reconstruction, posterior tibial slope, anterior cruciate ligament tear

## Abstract

Background

Increased posterior tibial slope (PTS) is an important risk factor for non-traumatic graft failure and revision surgery after anterior cruciate ligament reconstruction. If a tibial posterior slope is an important factor for graft failure after anterior cruciate ligament reconstruction, does it affect clinical outcomes? This study aimed to evaluate the association between PTS and clinical outcomes after anterior cruciate ligament reconstruction.

Material and methods

Patients undergoing arthroscopic anterior cruciate ligament reconstruction with hamstring tendons in the clinic were evaluated retrospectively. Inclusion criteria were: patients with at least an 18-month follow-up period who were evaluated with the Tegner Lysholm scoring system, aged between 18 and 40 years, with only an anterior cruciate ligament rupture. PTSs were measured from the lateral radiographs of the knees. The patients were divided into two groups with a PTS of 10° or less.

Results

The mean Tegner Lysholm score was 86.8 ± 8.9. The mean PTS was 9.7° ± 1.5°. In total, 14 and 15 patients had a PTS of above 10° and below 10°, respectively. The mean age and follow-up time of patients were 28.5 ± 5.3 years and 24.6 ± 7.2 months in the group with a PTS of above 10° and 30.2 ± 5.3 years and 24.2 ± 5.18 months in the group with a PTS of below 10°, respectively. Tegner Lysholm scores were 88.2 ± 8.8 and 85.6 ± 9.1 in the group with values above 10° and below 10°, respectively. Statistically, there was no significant difference between the clinical outcomes of both groups.

Conclusion

PTS does not affect the clinical outcomes of patients who underwent arthroscopic anterior cruciate ligament reconstruction in the early period.

## Introduction

Arthroscopic anterior cruciate ligament (ACL) reconstruction is one of the most common orthopedic surgical procedures. There are defined reasons for revision surgery after ACL reconstruction. Age, sex, body mass index, joint laxity, inability to open the tunnels in the appropriate position, graft thickness, inadequacy of the materials used in graft fixation, and tibial posterior slope are some of these [1,2].

Recent studies have reported that increased posterior tibial slope (PTS) is an important risk factor for non-traumatic graft failure and revision surgery after ACL reconstruction [3,4]. Increased PTS causes anterior translation of the tibia during walking [5]. According to the results of the previous studies, a 1° increase in the medial tibial slope causes a 1.24-fold increase in graft failure, a 1° increase in the lateral tibial slope causes a 1.17-fold increase in graft failure, and the risk of graft failure is higher in patients with a lateral tibial slope of 10 degrees or more [6,7]. Thus, if the tibial posterior slope is an important factor for graft failure after ACL reconstruction, does it affect clinical outcomes?

The purpose of this study was to evaluate the association between PTS and clinical outcomes after ACL reconstruction. The PTS of the patients was determined according to the preoperative lateral knee radiographs, and the clinical outcomes of both groups were compared by dividing them into two groups: those below and above 10°.

## Materials and methods

This study was conducted at Bahcesehir University, VM Medicalpark Pendik Hospital. This study was approved by the ethics committee of the local institute (Decision No. 2021-12/06). All patients provided informed consent. In this study, 62 patients who underwent arthroscopic ACL reconstruction with hamstring tendons in the clinic were evaluated retrospectively between 2016-2018. The inclusion criteria were as follows: patients with at least an 18-month follow-up period who were evaluated with the Tegner Lysholm scoring system at the last control, aged between 18 and 40 years, with only ACL rupture, and who underwent lateral knee X-ray before surgery. Patients with a follow-up period of less than 18 months who were not evaluated with the Tegner Lysholm scoring system, aged under 18 years and over 40 years, with chondral and meniscal tears with ACL tears, and who did not undergo a lateral knee X-ray in the preoperative period were excluded from this study. Twenty-nine patients who met the inclusion criteria were included in this study. PTSs were measured from the lateral radiographs of the knees obtained preoperatively. The patients were divided into two groups with a PTS of 10°or less.

Surgical technique

All ACL reconstructions were performed using hamstring tendons as autografts. After diagnostic arthroscopy, the semitendinosus and gracilis tendons were harvested. Tendons were prepared in a four-strand style. The femoral tunnel was prepared using the anteromedial portal approach. The tibial tunnel was prepared using the arthroscopic control technique. The prepared autograft was passed through tunnels; femoral side fixation was performed with an endobutton; and tibial side fixation was performed with a bioabsorbable screw and staple.

Post-operative follow-up and physical therapy

The patients used an angle knee brace for the first two weeks of the post-operative period. Up to 90° of flexion of the knee was allowed. After the first two-week period, the use of angle-adjustable knee braces was terminated, and a physical therapy program was started to strengthen the quadriceps and increase the knee joint’s range of motion. The same physical therapy program was applied to all patients. Early and late complications among the patients were determined. Treatments for complications were arranged. Patients were evaluated with the Tegner Lysholm score of the final controls.

Measuring method of PTS

**Figure 1:**
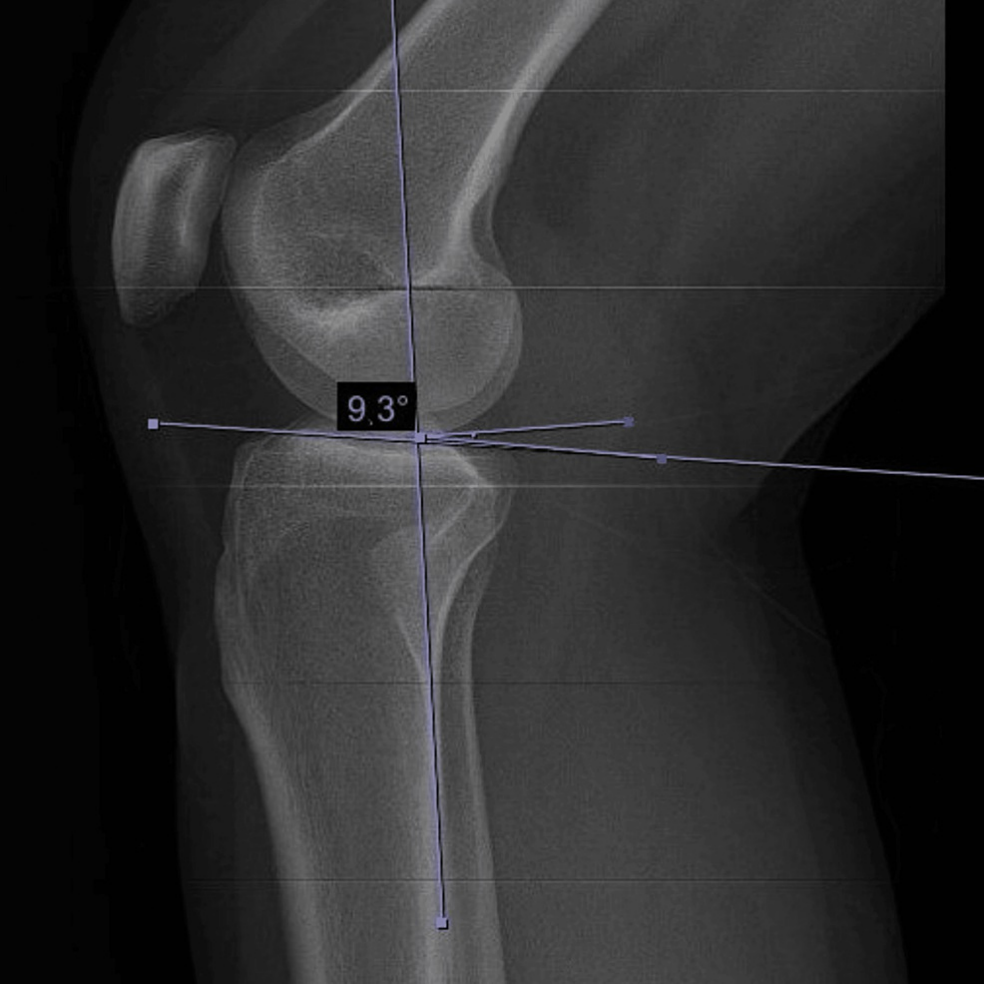
Measurement method of the posterior tibial slope

A line extending proximal to the tibial plateau was drawn at the posterior tibial cortex. The second line was drawn parallel to the medial tibial plateau. The angle between the two lines was measured. The measured angle was accepted as the posterior tibial slope [8] (Figure 1).

Statistical analysis

The suitability of the data for normal distribution was tested, and because they were not normally distributed, the non-parametric Mann-Whitney U test was used. A p-value <0.05 at the 95% confidence interval was considered statistically significant.

## Results

A total of 62 patients underwent ACL reconstruction with the hamstring tendon technique. There were 12 male (80%) and 3 female (20%) patients in group 1, and 11 male (78.6%) and 3 female (21.4%) patients in group 2. The mean follow-up durations were 24.6 ± 7.2 and 24.2 ± 5.1 months for groups 1 and 2, respectively. The mean ages of the patients were 28.5 ± 5.3 and 30.2 ± 5.3 years in groups 1 and 2, respectively. The mean BMIs were 27.2 ± 3.6 and 26.3 ± 3.8 kg/m2 in groups 1 and 2, respectively. There was no significant difference in age, BMI, or follow-up duration between the two groups (p>0.05).

The mean age of the patients in the study was 29.4 ± 5.3 years. The mean follow-up period was 24.4 ± 6.1 months. The mean Tegner Lysholm score was 86.8 ± 8.9. The mean PTS was 9.7° ± 1.5°. The lowest PTS was 7.8°, and the highest PTS was 12.5°. In total, 14 and 15 patients had a PTS of above 10° and below 10°, respectively. The mean tibial slope of the 1st group was 8.7° ± 0.9° (7.8°-9.1°), and that of the 2nd group was 11.1° ± 0.8° (10.2°-12.5°).

Tegner Lysholm scores were 88.2 ± 8.8 and 85.6 ± 9.1 in the group with a PTS above 10° and in the group with a PTS below 10°, respectively. Statistically, there was no significant difference between the clinical outcomes of both groups (p>0.48) (Table 1).

**Table 1:** Comparison of clinical outcomes of two groups Mean values of all patients included in the study and both groups. PTS: posterior tibial slope

	Number	The PTS (mean+SD)	Tegner Lysholm score	P-value
All patients	29	9.7° ± 1.5°	86.8 ± 8.9	
Patients with PTS above 10°	14	8.7° ± 0.9°	88.2 ± 8.8	p>0.48
Patients with PTS below 10°	15	11.1° ± 0.8	85.6 ± 9.1	

During the duration of the follow-up, revision surgery was not required at any time. In the early period, superficial cellulitis was found in two individuals, and antibiotic therapy was administered to those patients. No complications, including deep vein thrombosis and septic arthritis, were found during this examination.

## Discussion

The relationship between posterior tibial slope (PTS) and anterior cruciate ligament (ACL) injuries has garnered attention in recent studies, as PTS is believed to be a potential contributing factor to ACL injury risk. PTS is measured as the posterior inclinations of the medial and lateral tibial plateau, with typical values being 7° for the medial plateau and 5° for the lateral plateau [9]. It's hypothesized that an increased PTS can lead to greater knee rotatory flexibility, potentially increasing the risk of anterior tibial translation and, subsequently, ACL injury [10].

Several studies have explored the connection between PTS and ACL injuries. A meta-analysis reported an association between both medial and lateral PTS and ACL injuries [11]. However, another study found a statistically significant association only between lateral PTS and ACL injuries, not medial PTS [12]. Additionally, one study suggested that individuals with a PTS above 8° were at an increased risk of ACL injury [13]. Notably, while ACL injury has been associated with lateral posterior tibial inclination, a meta-analysis suggests that both medial and lateral posterior tibial inclination are linked to ACL injuries [11,14]. Moreover, patients with increased medial (>5.6°) and lateral (>3.8°) PTS have shown higher failure rates after primary ACL reconstruction using hamstring autografts in long-term follow-ups [15]. The measurement of the posterior tibial cortex is considered the most reliable method for analyzing PTS on lateral knee radiographs in patients undergoing ACL revision reconstruction [16]. However, in some studies, measurements of medial and lateral posterior tibial inclination were unavailable, making it challenging to assess their effects on clinical outcomes. In this study, medial and lateral posterior tibial inclination measurements could not be measured, so the effect of medial and lateral PTS on clinical outcomes could not be determined.

Various studies in the literature have reported different angle values as risk factors for ACL injury and the need for revision surgery after ACL reconstruction. For instance, some studies suggest that a PTS above 12° is associated with a higher risk of ACL injury and reconstruction failure [4,17,18]. Another study identified a PTS of ≥17° and anterior tibial translation of ≥6 mm as predictive risk factors for primary ACL reconstruction failure [19]. Additionally, a PTS above 10° is considered a risk factor for significant anterior tibial subluxation in the lateral compartment following non-contact ACL injuries [20].

The reasons for inadequacies after ACL reconstruction are multifactorial, including surgical technical errors, re-injury, and biological deficiencies [21]. High lateral tibial posterior slope can potentially lead to tunnel enlargement during ACL surgery [22], but osteotomies to reduce PTS in revision surgery have shown positive clinical outcomes [23,24]. Additionally, reducing the tibial posterior slope by more than 5° preoperatively has been reported to benefit the reconstructed ACL graft [25]. Correcting varus and posterior tibial tilt has led to decreased anterior tibial translation and reduced forces on the reconstructed ACL graft, supporting the goal of aligning the tibial plateau vertically, especially in ACL reconstruction surgery [26]. Based on Park et al.’s study, patients with ACL rupture who had successful results after conservative treatment had a lower mean posterior tibial curvature (8.3° in the successful group and 10.2° in the unsuccessful group) compared to those who failed conservative treatment. They have reported that the failure of conservative treatment after an ACL tear is associated with increased PTS [27].

This study is limited by the absence of a conservatively treated control group and the small number of participants.

## Conclusions

In conclusion, the posterior tibial slope has no effect on the clinical outcomes of patients who underwent arthroscopic anterior cruciate ligament reconstruction in the early treatment period. In the future, we anticipate that studies with longer follow-up periods and larger sample numbers will contribute to the existing body of literature.
